# Transcriptomic analysis and 3D bioengineering of astrocytes indicate ROCK inhibition produces cytotrophic astrogliosis

**DOI:** 10.3389/fnins.2015.00050

**Published:** 2015-02-20

**Authors:** Ross D. O'Shea, Chew L. Lau, Natasha Zulaziz, Francesca L. Maclean, David R. Nisbet, Malcolm K. Horne, Philip M. Beart

**Affiliations:** ^1^Department of Physiology, Anatomy and Microbiology, La Trobe UniversityBundoora, VIC, Australia; ^2^Florey Institute of Neuroscience and Mental Health, University of MelbourneParkville, VIC, Australia; ^3^Research School of Engineering, The Australian National UniversityCanberra, ACT, Australia; ^4^Department of Neurology, St. Vincent's HospitalFitzroy, VIC, Australia

**Keywords:** astrocyte, cytotrophic phenotype, Rho kinase, transcriptome, cell culture, bioengineering

## Abstract

Astrocytes provide trophic, structural and metabolic support to neurons, and are considered genuine targets in regenerative neurobiology, as their phenotype arbitrates brain integrity during injury. Inhibitors of Rho kinase (ROCK) cause stellation of cultured 2D astrocytes, increased L-glutamate transport, augmented G-actin, and elevated expression of BDNF and anti-oxidant genes. Here we further explored the signposts of a cytotrophic, “healthy” phenotype by data-mining of our astrocytic transcriptome in the presence of Fasudil. Gene expression profiles of motor and autophagic cellular cascades and inflammatory/angiogenic responses were all inhibited, favoring adoption of an anti-migratory phenotype. Like ROCK inhibition, tissue engineered bioscaffolds can influence the extracellular matrix. We built upon our evidence that astrocytes maintained on 3D poly-ε-caprolactone (PCL) electrospun scaffolds adopt a cytotrophic phenotype similar to that produced by Fasudil. Using these procedures, employing mature 3D cultured astrocytes, Fasudil (100 μM) or Y27632 (30 μM) added for the last 72 h of culture altered arborization, which featured numerous additional minor processes as shown by GFAP and AHNAK immunolabelling. Both ROCK inhibitors decreased F-actin, but increased G-actin labeling, indicative of disassembly of actin stress fibers. ROCK inhibitors provide additional beneficial effects for bioengineered 3D astrocytes, including enlargement of the overall arbor. Potentially, the combined strategy of bio-compatible scaffolds with ROCK inhibition offers unique advantages for the management of glial scarring. Overall these data emphasize that manipulation of the astrocyte phenotype to achieve a “healthy biology” offers new hope for the management of inflammation in neuropathologies.

## Introduction

Astrocytes make important contributions to the maintenance of the function of the mammalian central nervous system (CNS)—not only are they the most populous cells, but they play major roles in the maintenance of CNS health through their involvement in energetics, L-glutamate (Glu) homeostasis, anti-oxidant activity and release of trophic factors and gliotransmitters (Ridet et al., 1997; Maragakis and Rothstein, 2006; Parpura et al., 2012). Moreover astrocytes are well documented to be plastic cells that change their morphology, and hence biology, in response to alterations in the extracellular milieu, which may elicit short- and/or long-term responses. These morphological changes occur in normal and pathological brain tissue, and there is an ever expanding literature that astrocytes exist in diverse phenotypes across a continuum exhibiting pro-survival (“cytotrophic”) and destructive (“cytotoxic”) components (McMillian et al., 1994; Panickar and Norenberg, 2005; Sofroniew and Vinters, 2010). Astrocytes are historically considered to contribute to brain pathologies in a “secondary” mode during what has been termed reactive gliosis (Ridet et al., 1997; Maragakis and Rothstein, 2006), but there is now a solid body of growing evidence supporting their primary role in non-cell autonomous injury, where they secrete toxic entities and/or contribute to proteinopathies, driving disease progression in various neuropathologies (Lobsiger and Cleveland, 2007; Ilieva et al., 2009; Burda and Sofroniew, 2014). Astrocytes may contribute to non-cell autonomous injury in motor neurone disease (MND; amyotrophic lateral sclerosis) (Pirooznia et al., 2014) and other neurodegenerative conditions (Di Malta et al., 2012). Whilst the glial scar has long been considered a genuine target for drug development (Mueller et al., 2009), and especially the application of inhibitors of Rho kinase (ROCK) (Mueller et al., 2005), this view is simplistic given advances in our recent knowledge since astrocytes are much more than an inflammatory cell displaying adaptive plasticity in the functioning CNS. Thus many aspects of astrocyte biology offer options as attractive targets to improve their brain health and hence to effect a resultant improvement on synaptic function (Vargas and Johnson, 2010).

Our earlier research demonstrated the association between astrocytic morphology and a number of important aspects of astrocytic function, particularly the abundance and activity of Glu transporters (excitatory amino acid transporters, EAATs). We observed that altering the morphology of astrocytes, using cyclic AMP analogs or ROCK inhibitors, also increased Glu uptake (Lau et al., 2005), elevated transporter V_max_ with an approximate doubling of EAAT2 expression at the cell surface and a smaller increase in EAAT1 expression as quantified by biotinylation and immunoblotting (Lau et al., 2011). Similar changes in EAAT activity or abundance were also observed in other treatments altering astrocytic morhpoholgy (Zagami et al., 2005, 2009; O'Shea et al., 2006). We concluded that ROCK inhibitor-induced elevations in Glu transporter function may contribute to their beneficial actions in brain pathologies, since enhanced EAAT activity is likely to be beneficial in CNS injury where excitotoxicity is a common mechanism effecting neurodegeneration (Beart and O'Shea, 2007; Sheldon and Robinson, 2007). Later work led to the hypothesis that changes to the astrocytic cytoskeleton induced by Rho kinase inhibitors were accompanied by the adoption of a “healthy” phenotype. We defined this pro-survival, healthy phenotype as possessing elevated expression of EAAT2, BDNF and key anti-oxidant genes. A shift in the F-/G-actin ratio in favor of G-actin, indicating a reduction in actin stress fibers and alterations to cytoskeletal signaling mechanisms (Kuhn et al., 2000), was also considered integral to this “healthy” phenotype. Much more is known about ROCK inhibitors and their ameliorative actions on destructive (“cytotoxic”) glial scarring (Mueller et al., 2005), but our findings reveal diverse “healthy” effects on the astrocyte transcriptome likely to be beneficial in brain injury.

Our continued interest in the relationship between astrocyte morphology and biology led us to apply tissue engineering (Teo et al., 2006) to astrocytes. We found that 3D poly-ε-caprolactone (PCL) scaffolds altered astrocytic responses *in vivo* in a model of traumatic brain injury (Nisbet et al., 2009). Here our hypothesis was promotion by the bioscaffold of a cytotrophic astrocytic phenotype, so when considered with a likely role for the extracellular matrix (ECM) (Lau et al., 2012), we speculated about links to Rho GTPases, perhaps involving the actin cytoskeleton. In primary culture, astrocytes on 3D PCL scaffolds displayed reduced cytoskeletal stress as confirmed by decreased expression of GFAP and increased G-actin (Lau et al., 2014), and, when maintained over an extended periods, possessed an extensively arborized, stellate morphology. These astrocytes showed a gene expression profile strikingly similar to that of 2D astrocytes treated with Fasudil, with up-regulation of genes for EAAT2, BDNF and anti-oxidant enzymes (Lau et al., 2014). Since 2D astrocytes treated with Rho kinase inhibitors also adopt a stellate shape, our astrocyte transcriptome (Lau et al., 2012) is likely to contain insights into previously unsuspected mechanisms given new literature on this class of molecules.

In this study, we sought to place our findings in their contemporary context (Parpura et al., 2012; Burda and Sofroniew, 2014), by further interrogating our transcriptome after Fasudil treatment through mining this astrocytic database to reveal previously unexplored biological themes. Secondly, given our success with 3D bioengineered astrocytes, we undertook additional analyses on the possible combined benefits of Rho kinase inhibitors in our 3D culture model. Together these data provide further evidence that ROCK inhibitors produce physiologically beneficial responses in astrocyte biology which are likely to be beneficial in the management of inflammation in diverse neuropathologies.

## Materials and methods

### Animals

C57BL/6 mice were obtained from the Florey Neuroscience Institutes (Melbourne, VIC, Australia). All experiments receive ethical approval from the Florey Neuroscience Institutes Animal Experimentation Ethics Committee (ethics approval number 07-061). Experiments were performed in accordance with the Prevention of Cruelty to Animals Act 1986 under the guidelines of the National Health and Medical Research Council Code for the Care and Use of Animals for Experimental Purposes in Australia.

### Bioengineering, cytochemistry and neurochemical assays

Secondary astrocytic cultures were established from forebrain of postnatal d1.5 mice as described previously (Lau et al., 2011).

Briefly, forebrains were dissected in ice-cold solution (HBSS, Hanks balanced salt salution: 137 mM NaCl, 5.37 mM KCl, 4.1 mM NaHCO_3_, 0.44 mM KH_2_PO_4_, 0.13 mM Na_2_HPO_4_, 10 mM HEPES, 1 mM sodium pyruvate, 13 mM D(+)glucose, 0.01 g/L phenol red), containing 3 mg/ml bovine serum albumin (BSA) and 1.2 mM MgSO_4_, pH7.4). Cells were dissociated, centrifuged, and the pellet resuspended in astrocytic medium (AM: DMEM, Dulbecco's modified eagle medium, 10% FBS, 100 U/ml penicillin/streptomycin, 0.25% (v/v) Fungizone™), preheated to 36.5°C at a volume of 5 ml per brain and plated at 10 ml per 75 cm^2^ flask. Cells were maintained in a humidified incubator supplied with 5% CO_2_ at 36.5°C and complete medium changes were carried out twice weekly.

After 10 *days in vitro* (*div*), when a confluent layer had formed, the cells were shaken overnight (180 rpm) and rinsed in fresh medium to remove non-astrocytic cells. Astrocytes were subsequently detached using 5 mM EDTA (10 min at 37°C) and seeded on 96-well plates, random or aligned PCL scaffolds in 96-well plates (all 8 × 10^3^ cells/well) or on 13 mm glass coverslips (conventional 2D controls) in 24-well plates (2 × 10^4^ cells/well). 3D fibrous scaffolds were engineered from unfunctionalized PCL using electrospinning (Nisbet et al., 2009); thicknesses were approximately 250 and 150 μm for random and aligned scaffolds, respectively. Astrocytes were treated 8 *div* later with vehicle, N^6^,2′-O-dibutyryladenosine 3′,5′-cyclic monophosphate (dbcAMP,1 mM), or Rho kinase inhibitors Y27632 (30 μM) or Fasudil (100 μM) for a further 72 h when biochemical and morphological analyses were undertaken.

Cytochemistry for GFAP, F-actin and G-actin has been described previously (Lau et al., 2011). For immunocytochemistry, cells were washed with phosphate buffered saline (PBS:137 mM NaCl, 0.5 M Na_2_HPO_4_, 0.5 M NaH_2_PO_4_, pH 7.4) and fixed in 4% paraformaldehyde (PFA) in PBS for 10 min, followed by three washes with Tris buffered saline (TBS: 50 mM Tris–HCl, 1.5% NaCl, pH 7.6). Non-specific binding was blocked with 10% normal goat serum/normal donkey serum (NGS/NDS) in TBS containing 0.3% Triton X-100. Cells were then incubated with primary antibodies against GFAP (1:1000; Chemicon) or AHNAK (1:500; Molecular Probes), a marker of enlargeosome activity (Racchetti et al., 2012), at 4°C overnight on a rocker platform. Cells were then washed and incubated with secondary antibodies (anti-rabbit Alexa Fluor® 488 for GFAP 1:500; anti-mouse Alexa Fluor®568 for AHNAK 1:500; Molecular Probes) and Hoechst 33342 (1:500 dilution) diluted in 2% (v/v) NGS or NDS in PBS containing 0.3% (v/v) Triton X-100 for 3 h at room temperature. Cells were again washed with PBS three times at room temperature. Coverslips and scaffolds were then mounted on glass microscope slides using Dako fluorescence mounting medium and left to dry in the dark overnight. Both coverslips and scaffolds were stored at 4°C until examined by microscopy.

For concurrent labeling of F- and G-actin, cells were washed rapidly with PBS twice by vacuum aspiration and incubated in stabilizing solution (10 mM Tris base, 0.15 M NaCl, 0.01% Triton X-100, 2 mM MgCl_2_, 0.2 mM DTT (Bio Vectra, Canada), 10% glycerol) for 1 min at 4°C. Cells were then washed rapidly with chilled PBS (4°C) twice and fixed in 4% (v/v) PFA in PBS for 15 min at 4°C. Cells were washed in PBS twice at room temperature and excess PFA was quenched by adding 50 mM NH_4_Cl in PBS for 15 min at room temperature. Cells were permeabilized in 0.5% (v/v) Triton X-100 in PBS for 5 min and incubated in blocking solution (2% (v/v) BSA, 0.1% (v/v) Triton X-100 in PBS) for 15 min at room temperature. Following another two washes with PBS, cells were incubated in dye solution (DNaseI-Alexa Fluor 488 1:250; Molecular Probes; rhodamine-phalloidin 1:125 in blocking solution; BDH) for 30 min in the dark. Cells were then washed in PBS three times and mounted on glass microscope slides using Dako fluorescence mounting medium and left to dry in the dark. Cells on scaffolds were mounted by placing the scaffolds with the cells on top, mounted with Dako fluorescence mounting medium and glass coverslips (13 mm round; Menzel-Glaser). Slides were stored at 4°C until required for imaging.

Methods for measurement of cellular viability [3-(4,5-dimethylthiazol-2-yl)-2,5-diphenyltetrazolium bromide (MTT; an index of mitochondrial function) and lactate dehydrogenase assays] have been published (Lau et al., 2011). After treatmentd, MTT was added to the wells give a final concentration of 0.5 mg/ml, incubated with the cells at 36.5°C with 5% CO2 for 30 min. Media were aspirated and 300 μl of dimethyl sulfoxide (DMSO) was added into each well to dissolve the formazan product. The absorbance was subsequently measured at 570 nm using a Bio-Rad Benchmark Plus microplate spectrophometer. Lactate dehydrogenase (LDH) assay was carried out using a commercially available kit (Roche). Medium (50 μl) was collected from each well and placed in a 96-well plate. The samples were incubated with the reaction mixture (Cytotoxicity Detection Kit from Roche) according to the manufacturer's protocol and left in the dark for 30 min. The absorbance of the sample mixture was determined at 490 nm using a Bio-Rad Benchmark Plus microplate spectrophometer. Data from these experiments were analyzed using Two-Way repeated-measures ANOVA with Bonferroni's *post-hoc* test using Graphpad Prism software (Version 6).

To examine changes in relative abundance of F-actin and G-actin, the G-actin image was “subtracted” from the corresponding F-actin image after both images were converted to gray-scale, and integrated optical density was measured using ImageJ (NIH: version 1.37). Image analysis used data from 4 images/well from 2 wells/culture over 3 independent cultures. The average value for all fields subjected to the same treatment in an individual experiment was analyzed as a single data point. All images for each form of actin were obtained using the same exposure settings. Statistical comparisons were made using Two-Way repeated-measures ANOVA with Bonferroni's post-hoc test using Graphpad Prism software (Version 6).

### Microarray analyses

Full details have been given previously (Lau et al., 2012), where we validated microarray data by quantitative RT-PCR. Differentially expressed genes between control and Fasudil-treated samples at each time point were then filtered to include only those passing a stringent false cut off of 0.05. Data have been previously deposited in NCBI's Gene Expression Omnibus and are accessible through GEO Series accession number GSE25829 (Lau et al., 2012).

## Results

### Novel insights from transcriptomic profiling into beneficial actions of Fasudil in astrocytes

Our initial rationale for undertaking microarray analyses to define the genomic changes induced by Fasudil in astrocytes was the total “disconnect” between the extremely rapid changes in astrocytic shape, which were quite obvious at 30 min, and the alterations in Glu transport which were of a much slower time course (≥24 h) (Lau et al., 2011, 2014). We reasoned that appreciable transcription and new protein synthesis must be taking place to underpin these large changes, and that understanding the molecular changes in astrocytes should allow new mechanistic insights into how ROCK inhibitors provide benefit during brain insults. Our initial bioinformatics revealed that differentially expressed genes at 2 and 6 h were predominantly down-regulated, and after gene ontology analysis, did not appear to follow any particular biological theme so our focus was on large significant fold changes at later time points (12 and 24 h). Our attention thus settled upon major biological processes regulating astrocytic motility and cytoskeletal reorganization viz. actin cytoskeleton, axon guidance, transforming growth factor-ß signaling and tight junctions. We also found large changes in many genes associated with the ECM (Lau et al., 2012). Here, in view of new understanding that astrocytic responses occur across a continuum that is dependent upon the extent of trauma/disease, and which may resolve when minor and be manageable even in glial scarring by pharmacological intervention (Mueller et al., 2009; Sofroniew, 2009), we undertook new mining of our transcriptomic database accessible through GEO Series accession number GSE25829 (Lau et al., 2012).

#### Transport and molecular motors

Since our published work had ended with a focus on the pro-survival (“cytotrophic”) astrocytic phenotype produced by ROCK inhibition (Lau et al., 2012), we took a step back and focused our attention on mechanistic issues related more broadly to signaling and trafficking events underpinning astrocytic motility and cytoskeletal reorganization. ROCK inhibitors effect disassembly of actin stress fibers and focal adhesions (Mueller et al., 2005), and we documented rapid dissipation (as early as 15 min) of phalloidin-labeled actin stress fibers in cultured murine astrocytes treated with Fasudil. Here we demonstrated the actin cytoskeleton underwent a transition to a preponderance of G-actin relative to F-actin, which were increased and decreased 4-fold, respectively (Lau et al., 2010). Rho GTPases, notably Rho and Rac, are key regulators of actin and microtubule cytoskeletons, and actin flow can regulate the positioning of the microtubule cytoskeleton. Active transport, be it antero- or retro-grade, plays a key role in the delivery of gene products and cellular organelles and has been studied in detail in neurones—its disruption leads to “transportopathies” in various neurodegenerative conditions (Liu et al., 2012). Very little is known of these events in astrocytes where we found expression profiles of kinesin family members (*KIF2A*, *KIF13A*, *KIF 18A*, and *KIF21*), involved in anterograde transport, were down-regulated (2-3 fold) at 2 and 6 h after Fasudil (*KIF2A*, Table 1). Dynein and dynactin members linked to the retrograde motor system also displayed reduced expression at early time points but had returned to control by 24 h. We extended these analyses to include GTPase Rabs, which act as molecular switches to mediate vesicular transport along the cytoskeleton by engaging specific motor proteins (Ng and Tang, 2008)—member of RAS oncogene family 3 (RAB3) may play a role in exocytosis in astrocytes and there was a notable down-regulation of the expression of its isoform *RAB3D* at 12 and 24 h (Table 1). Interestingly two targets of ROCK phosphorylation, syntaxin binding protein 1A (*STX1A*) involved in vesicle docking/fusion (3-fold decrease, 6 h), and dystrophin related protein 2 (*DRP2*, collapsing response mediator protein 2; 2-fold decreases, 12 and 24 h; Table 1) linked to semaphorin-mediated guidance mechanisms (Arimura et al., 2000), were also down-regulated. Given the general trend of data here after ROCK inhibition was decreased gene expression, we wondered whether the consequent stellation with astrocytes adopting an aligned linear and a pro-survival phenotype (Lau et al., 2011, 2012) might also reflect transition to a non-migratory state as has been suggested in normal brain (Cárdenas et al., 2014).

**Table 1:** **Selected genes with expression changes passing the filter of fold change >2.0 and FDR <0.05 in at least one time point for Fasudil-treated compared with untreated astrocytes**.

**Probeset ID**	**Definition**	**2 h**		**6 h**		**12 h**		**24 h**	
		***P*-value**	**Fold-change**	***P*-value**	**Fold-change**	***P*-value**	**Fold-change**	***P*-value**	**Fold-change**
KIF2A	Kinesin family member 2A	*8.89E-03*	−1.48	*1.34E-07*	−2.89	1.89E-01	1.20	8.36E-01	−1.03
RAB3D	Member RAS oncogene family	8.14E-02	1.23	*7.50E-03*	1.40	*1.76E-07*	−2.37	*1.55E-05*	−1.88
DRP2	Dystrophin related protein 2	*2.06E-02*	1.53	5.92E-01	1.10	*5.41E-05*	−2.35	*4.62E-03*	−1.71
FOXO1	Forkhead box O1	*1.99E-09*	−4.19	*2.62E-05*	−2.16	*1.96E-04*	1.91	*4.26E-02*	1.36
SQSTM1	Sequestosome 1	*1.43E-03*	−1.36	*8.35E-09*	−2.17	*1.04E-02*	1.27	*2.03E-04*	1.46
MFN1	Mitofusin 1	4.32E-01	1.17	*7.11E-06*	−3.20	3.32E-01	−1.22	2.61E-01	1.26
JAK2	Janus kinase 2	*3.59E-02*	−1.34	*1.29E-05*	−2.10	2.78E-01	1.16	2.71E-01	1.16
NFKB1	Nuclear factor of kappa light polypeptide gene enhancer in B-cells 1	*1.24E-06*	−1.85	*1.33E-03*	1.40	*2.26E-03*	1.37	2.67E-01	1.11
SOCS3	Suppressor of cytokine signaling 3	*4.67E-08*	−5.41	5.77E-02	−1.51	3.87E-01	1.20	4.94E-01	−1.15
HIF1A	Hypoxia inducible factor 1, alpha subunit	6.84E-01	1.07746	*3.97E-02*	−1.486	1.39E-01	1.32015	4.31E-01	1.15591
VEGFA	Vascular endothelial growth factor A	*6.18E-06*	−3.49	8.83E-02	−1.45	2.28E-01	1.30	5.78E-01	1.13
HK2	Hexokinase 2	*4.25E-07*	−4.96	*2.95E-05*	−3.25	3.48E-01	−1.24	1.92E-01	−1.35

*Gene expression data are from NCBI's Gene Expression Omnibus and are accessible through GEO Series accession number GSE25829 (Lau et al., 2012). The FDR threshold of 0.05 corresponds to a P-value of 5.2 × 10^−3^, 01.47 × 10^−2^, 5.55 × 10^−3^ and 1.72 × 10^−3^ for the 2, 6, 12, and 24 h time points, respectively. P-value passing the FDR threshold at each time point are italicized*.

#### Autophagic and lysososmal systems

Autophagy, a key system regulating cellular homeostasis including protein and organelle degradation, is known to be affected by ROCK inhibitors, and is also considered a novel target for management of neurodegenerative diseases (Harris and Rubinsztein, 2012). Indeed, actions here would hardly be surprising given the cytoskeletal changes and the fact autophagosomes themselves are membraneous cargo moving along microtubules and transported by the kinesin and dynein/dynactin complex—expression of genes related to molecular motors was generally found to be decreased here. ROCK1/2 regulate actin dynamics and cell migration through phosphorylation of various substrates, but little is known as to how the cytoskeleton influences the activity of the ubiquitin proteasome system and autophagy. ROCK inhibitors have system-dependent actions being reported to accelerate autophagic flux (Mleczak et al., 2013), enhance both UPS and autophagic activity (Bauer et al., 2009), and to inhibit autophagosome formation (Aguilera et al., 2012). The literature on autophagy in astrocytes is relatively small (Dello Russo et al., 2013), but suggestive that as highly plastic glia they adapt to stress and support neurones (Lee et al., 2010; Titler et al., 2013). Mammalian forkhead members of the class O (FOXO) are master signaling integrators influencing many cellular responses, including oxidative stress and inflammation. FOXO pathways are linked to both autophagy and the UPS, and lack of FOXO1 prevents autophagy (Yang et al., 2013). While there is a substantial literature on the role of *FOXO1* in inflammation, very little is known about its involvement in astrogliosis. However, recently oxidative-induced injury of astrocytes was reported to involve metallothionein-3 via a FOXO-dependent mechanism (Lee et al., 2014). In our database, substantial reductions were found in the expression of *FOXO1* at 2 and 6 h (4- and 2-fold, respectively) post-Fasudil, although significant changes were not observed at longer times (Table 1). We extended our examination to other autophagic genes and found the expression of sequestosome 1 (*SQTM1*, p62), a multifunctional scaffolding/adaptor protein interacting with both autophagosomal and proteosomal systems (Korolchuk et al., 2010), was also decreased at both 2 and 6 h (Table 1). Mitochondrial transport is also driven by molecular motors with adaptor proteins linking mitochondria to microtubule-based transport, and there is a rapidly expanding literature on mitochondrial dynamics and recruitment of mitophagy, an unique form of autophagy handing damaged mitochondria (Baker et al., 2014). Expression of mitofusin 1 (*MFN1*), an outer mitochondrial membrane protein involved in mitochondrial dynamics, was down-regulated 3-fold at 6 h (Table 1), and had returned to control levels at longer time intervals. Ras homolog gene family members T1/2 (*RHOT1/2*, Miro1/2), Rho GTPases mediating mitochondrial transport by sensing [Ca^2+^], also underwent two-fold decreases in expression at 6 h. Many genes involved in mitochondrial dynamics, including fission and fusion, contribute to the pathological events in brain pathologies (Wang et al., 2009; Baker et al., 2014). Overall inhibition of Rho kinase results in an astrocyte where protein degradation pathways appear down-regulated initially and then assume a relatively inactive mode consistent with adoption of a healthy, anti-migratory phenotype.

#### Pro-inflammatory mechanisms

Inflammatory events related to glial scarring have received attention in the earlier literature (Mueller et al., 2005; Ding et al., 2010; Yu et al., 2010), so we examined these events in our astrocytic system after ROCK inhibition. Unregulated activation of the Janus Kinase-Signal Transducer and Activators of Transcription (JAK-STAT) pathway is a key driver of various inflammatory conditions and has been identified as a target for therapeutic intervention (Kaminska and Swiatek-Machado, 2008). Although less understood in brain, oxidative stress and some cytokines activate via a JAK2-dependent mechanism STAT3 (Planas et al., 2006). Numerous changes, mainly at early time-points, were noted in these pro-inflammatory mechanisms and were suggestive of decreased activity. Although down-regulation of expression of *JAK2* was found at 2 and 6 h (Table 1), interestingly in our mature cultured astrocytes the expression of *STAT3* (data not shown) was unchanged by Fasudil. Although recent elegant work points to a quite precise role of *STAT3* in scar-forming astroglia surrounding inflammatory cells in spinal cord injury (Wanner et al., 2013), the recruitment of astrocyte cellular signaling in inflammation appears context dependent (Sofroniew, 2014). We found that the expression of both nuclear factor of kappa light chain gene exchanger in B cells (*NFKB1*) and suppressor of cytokine signaling (*SOCS3*) was reduced at 2 h and had returned to essentially control levels at longer time intervals (Table 1). While *NFKB* deletion or knockdown reduces inflammation in a number of CNS injury models (Sofroniew, 2014), the regulation of SOCS system is extremely complex generally functioning to reduce chronic inflammation (Linossi et al., 2013). Indeed conditional ablation of SOCS3, but not STAT3, produces contraction of lesion area and notable improvement in functional recovery after spinal cord contusion (Okada et al., 2006).

#### Hypoxic-inducible factor-1 system and angiogenesis

We previously characterized the hypoxic-inducible factor-1 (HIF-1) system in an astrocytic model of tolerance against oxidative injury where there was downstream production of vascular endothelial growth factor (VEGF) (Chu et al., 2010). Thus herein we were interested to explore the HIF-1 system and the effects on downstream genes involved in angiogenesis and energetics, since these mechanisms are potentially neuroprotective (Trendelenburg and Dirnagl, 2005). Astrocytes are the major source of brain VEGF in the brain and various stimuli can modulate its induction and secretion (Engelhardt et al., 2014). Whilst inhibition of ROCK can activate VEGF-driven neovascularization and angiogenesis (Kroll et al., 2009), it is very clear the VEGF-mediated responses are concentration- and system-dependent being either beneficial or detrimental in the brain (Ellison et al., 2013). The expression levels of *VEGFA* and *HIF1A* were significantly reduced at 2 and 6 h, respectively, and had returned to control levels at longer time intervals (Table 1); significant changes were not found for erythropoietin (data not shown). Whilst a small body of evidence pertinent to astrocytes indicates that HIF1A regulates downstream expression of VEGF (Chavez et al., 2006; Chu et al., 2010), we were surprised to find a large 3-fold decrease of *VEGFA* at 2 h, evidence which might support the recently described regulation of VEGFA expression independent of HIF1 signaling in astrocytes (Arany et al., 2008; Schmid-Brunclik et al., 2008). Recently, astrocyte-derived VEGFA was reported to drive blood-brain barrier disruption (where astrocytes also retract their endfeet from vessels) in brain inflammatory disease and inhibition of VEGFA signaling suggested as a protective approach (Argaw et al., 2012). Astrocytes are less susceptible than neurones to injury by impairment of oxidative metabolism, at least in part because of their capacity to switch to glycolysis, which particularly under conditions of hypoxia is linked to the HIF-1 system (Schmid-Brunclik et al., 2008). Here hexokinase 2 (*HK2*), a key glycolytic enzyme, considered an integral component of the downstream response displayed large reductions in expression at both 2 and 6 h consistent with the *VEGFA* data (Table 1).

### ROCK inhibitors provide additional benefits for astrocytes on 3D electrospun scaffolds

#### Effects of drug treatments on astrocytes cultured on glass coverslips

**Figure 1:**
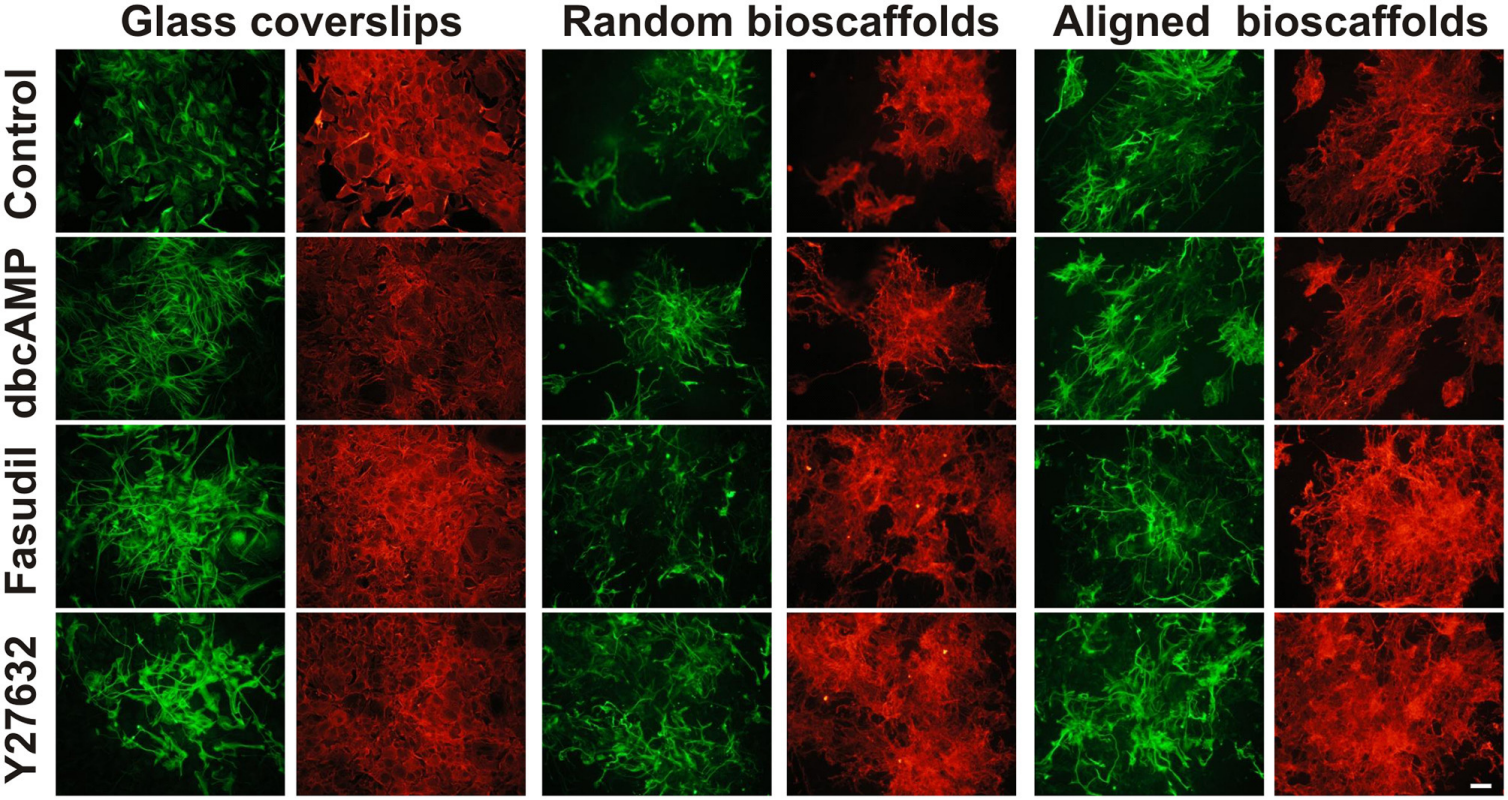
**Effect of drug treatments on morphology and expression of GFAP and AHNAK in astrocytes cultured on different substrates**. Astrocytes were treated for 72 h with vehicle (Control), dbcAMP (1 mM), Fasudil (100 μM) or Y27632 (30 μM) on glass coverslips, random or aligned bioscaffolds and immunostained to reveal GFAP (green) or AHNAK (red). Paired images represent the same field. Scale bar = 50 μm.

Cultures of mouse astrocytes were established on glass coverslips (2D) or on random or aligned PCL scaffolds for 18 *div* and immunolabeled with antibodies against GFAP and AHNAK. In agreement with previous studies, conventional 2D astrocytes exhibited a more stellate morphology with more extensive processes when treated for 3 days with dbcAMP (1 mM), Fasudil (100 μM) or Y27632 (30 μM) (Figure 1) (c.f. Lau et al., 2012). Under control conditions, 2D astrocytes appeared as flattened, polygonal cells and most were GFAP positive. Labeling for AHNAK immunocytochemistry was more widespread and partially co-localized with GFAP under conventional conditions (see below; Figure 1). When treated with dbcAMP, astrocytes appeared to undergo complete stellation, with reduced cell body area and elongated processes. The processes were thicker and shorter when compared to astrocytes treated with both ROCK inhibitors. Interestingly, the staining for AHNAK in astrocytes treated with dbcAMP was relatively darker compared to control (Figure 1). The effects of Y27632 treatment on astrocytes mimicked that of Fasudil. Cells demonstrated increased retraction of cell bodies as well as elongated extensive processes. Labeling patterns for AHNAK, a marker of enlargeosomes (Racchetti et al., 2012), were similar to GFAP distribution, but more widespread under control conditions and following experimental treatments (Figure 1). Notably in all cases GFAP-positive processes were well defined whereas AHNAK labeling of astrocytes was more intense and its distribution through all parts of the astrocytic arbor, including fine processes, made full resolution difficult.

#### Effects of bioscaffolds on astrocytic morphology

Astrocytes displayed a different phenotype when cultured on either random or aligned bioscaffolds with cells possessing elongated cell bodies, ramified cell processes and condensed GFAP filaments (Figure 1). On random scaffolds, astrocytes formed tighter clusters, approximately 100–250 μm in diameter, but use of aligned bioscaffolds produced astrocytes with more extensive elongated processes (approximately 50–250 μm) following fiber orientation that were distributed in loose clumps. Under control conditions, the labeling pattern for GFAP was found to partially co-localize with AHNAK, although the latter was more widespread, in cultures on both biomatrices, similar to those cultured on glass coverslips (Figure 1).

#### Effects of drug treatments on astrocytes cultured on different substrates

Astrocytes treated with dbcAMP (1 mM, 72 h) appeared to form clusters on both random (clusters approximately 100–200 μm diameter) and aligned scaffolds (approximately 75–125 μm diameter). In contrast to 2D cultured astrocytes, the processes were extensive, and outgrowth of the processes was more widespread. Processes infiltrated both type of scaffolds, as previously described (Lau et al., 2014) in random scaffolds (process length approximately 75–150 μm), but there was more growth along aligned fibers (approximately 100–300 μm) (Figure 1). As was the case in astrocytes cultures on glass coverslips, immunolabeling of AHNAK was more widespread and more ubiquitously expressed through the entire astrocytic arbor than GFAP with which it was generally co-localized in major processes (Figure 1).

When treated with Fasudil (100 μM, 72 h) astrocytes were evenly distributed and infiltration into both biomatrices was extensive. The processes appeared to be elongated approximately 50–250 μm on random scaffolds and 100–300 μm on aligned scaffolds, with retracted cell bodies. The process outgrowth was widespread on random bioscaffolds, but astrocytes on aligned bioscaffolds appeared to grow in a similar orientation as those on random scaffolds, and were evenly distributed forming less tight clusters with approximate 200–350 μm in diameter on both random and aligned scaffolds (Figure 1). Microscopic examination revealed that the labeling pattern for AHNAK was more widespread through the whole arbor than that of GFAP, which was restricted to major processes, on both biomatrices (Figure 1).

The effects of the other Rho Kinase inhibitor, Y27632, mimicked that of Fasudil. Astrocytes treated with Y27632 (30 μM, 72 h) also demonstrated extensive processes on both types of scaffolds, however the processes appeared to be longer than in astrocytes treated with Fasudil, with an approximate 150–300 μm in astrocytes cultured on random and approximately 75–350 μm length in astrocytes cultured on aligned biomatrices (Figure 1). AHNAK immunolabeling revealed much more of the astrocytic arbor and thus was partially co-localized to GFAP but more widespread.

#### Effects of drug treatments on actin expression

**Figure 2:**
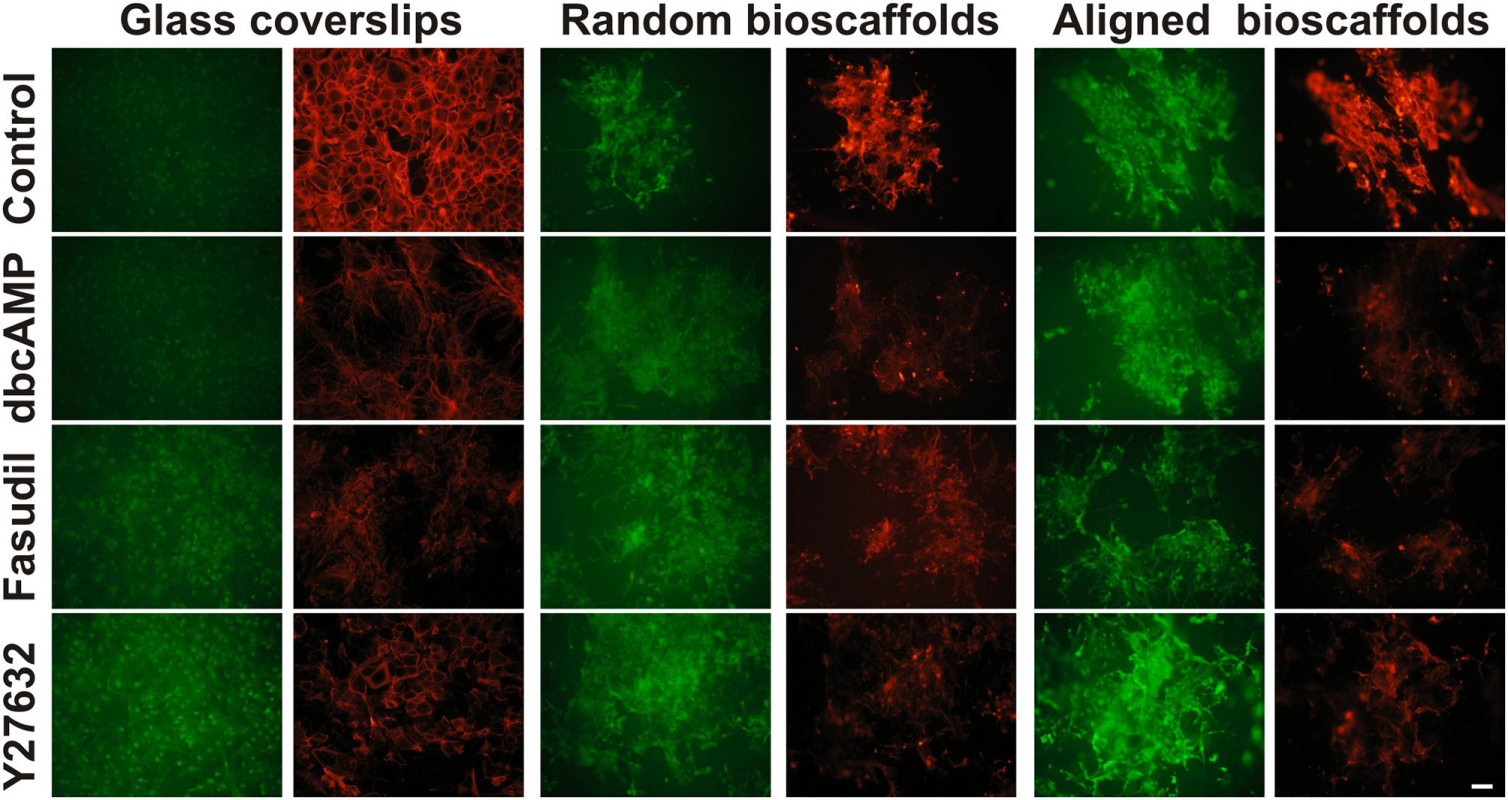
**Effect of drug treatments on expression of G-actin and F-actin in astrocytes cultured on different substrates**. Astrocytes were treated for 72 h with vehicle (Control), dbcAMP (1 mM), Fasudil (100 μM) or Y27632 (30 μM) on glass coverslips, random or aligned bioscaffolds and labeled to reveal G-actin (green) or F-actin (red). Paired images represent the same field. Scale bar = 50 μm.

Since the actin cytoskeleton plays a determinant role in regulating cellular responses to the extracellular matrix, the effects of fibrillar surfaces on actin dynamics were examined by staining astrocytes for its two forms: F-actin (rhodamine-conjugated phalloidin) and G-actin (Alexa Fluor 488-conjugated DNaseI) (Figure 2). Under control conditions 2D astrocytes displayed well-organized F-actin fibers with densely packed stress fibers and a diffuse expression of globular G-actin. When treated with dbcAMP for 72 h, the intensity of labeling for G-actin increased, while F-actin displayed a prominent change from well-organized actin rings packed with stress fibers to more elongated processes with reduced stress fibers. A similar pattern of changes was found in astrocytes treated with the Rho kinase inhibitors Fasudil and Y27632, where remodeling of the actin cytoskeleton was demonstrated by a shift from F-actin to G-actin predominance. F-actin in astrocytes treated with Y27632 exhibited the same morphology as control, displaying a well-organized ring shape without stress fibers. In contrast, F-actin formed clusters and exhibit a more globular shape in astrocytes treated with Fasudil.

When cultured on random bioscaffolds, F- and G-actin appeared to clump in the absence or presence of drug treatments. Astrocytes treated with dbcAMP and Fasudil displayed wider clumping of both F- and G-actin in relative to control, while cells treated with Y27632 appeared in tighter clusters (Figure 2). On aligned scaffolds, astrocytes formed clumps in the absence or presence of drug treatments. Additionally, under control conditions or following treatment with Y27632, both F-/G-actin clumps appeared to be more “rectangular” in shape, with some increase in longer processes, presumably aligned with the fibers (Figure 2). Astrocytes treated with either dbcAMP or Fasudil formed F-/G-actin in tight small clusters, however F-/G-actin in astrocytes treated with Fasudil displayed elongated processes compared to cells treated with dbcAMP. There was maintenance of overall G-actin labeling under all conditions, and notably with Y27632, whereas all treatments decreased F-actin relative to control (Figure 2).

**Figure 3:**
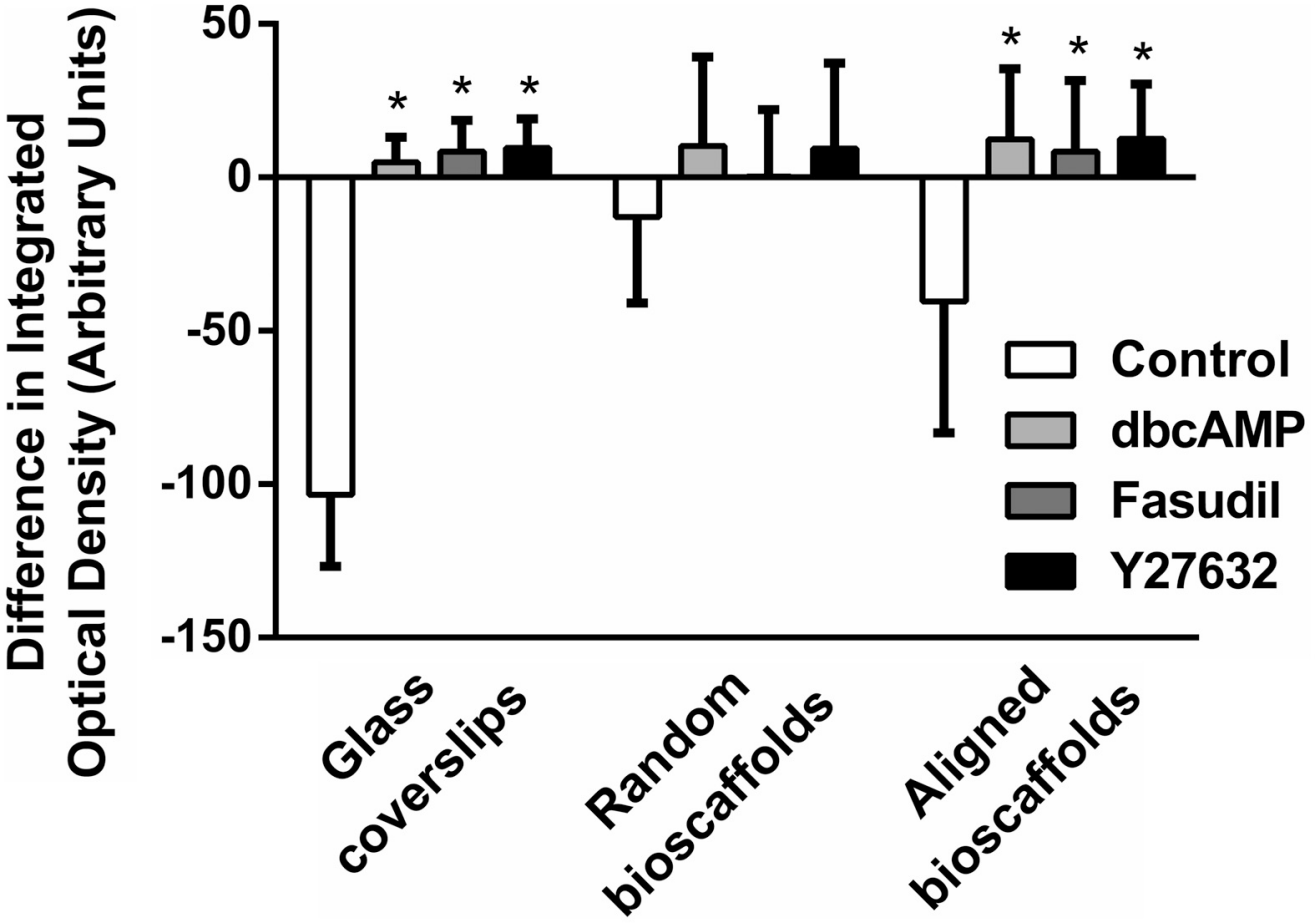
**Image analysis of difference in integrated density for F-/G- actin ratio**. Astrocytes were treated for 72 h with vehicle (Control), dbcAMP (1 mM), Fasudil (100 μM) or Y27632 (30 μM) on glass coverslips, random bioscaffolds or aligned bioscaffolds. ^*^Significantly different from control on same substrate, *p* < 0.05. Comparisons were made using Two-Way repeated measures ANOVA with Bonferroni's *post-hoc* test. Data represent mean ± S.E.M. *n* = 6 (duplicates from 3 individual experiments).

Image analysis revealed a significant difference in integrated optical density, reflecting a shift from F-actin to G-actin predominance when astrocytes were cultured in 2D or aligned fibers (*P* < 0.05 for all treatments versus Control) after 72 h treatment (Figure 3). A similar pattern of F-/G-actin ratio shift was observed in astrocytes cultures on random scaffolds but this change was not statistically significant.

#### Effects of drug treatments on cell viability

**Figure 4:**
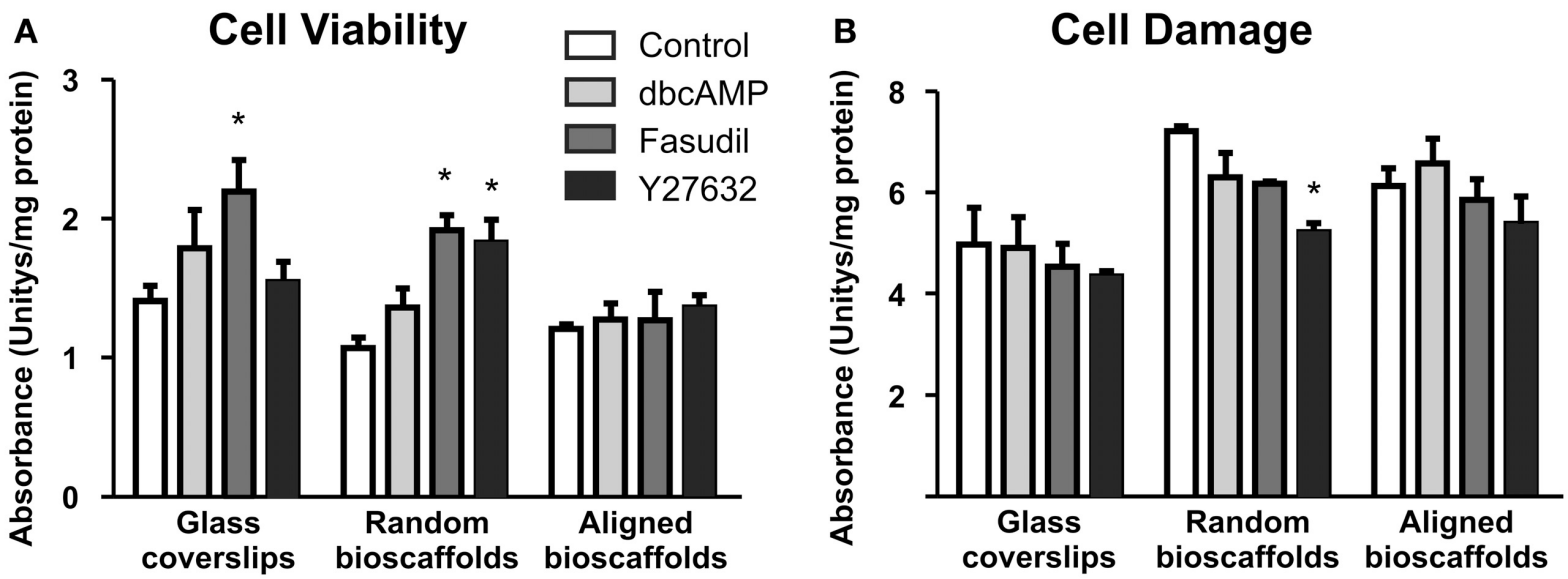
**Effect of drug treatments on cell viability and cell damage**. Astrocytes were treated for 72 h with vehicle (Control), dbcAMP (1 mM), Fasudil (100 μM) or Y27632 (30 μM) on glass coverslips, random or aligned bioscaffolds. Cell viability was determined by the 3-(4,5-dimethylthiazol-2-yl)-2,5-diphenyltetrazolium bromide assay **(A)**, while cell damage was assessed using the lactate dehydrogenase assay **(B)**. ^*^Significantly different from control on same substrate, *p* < 0.05. Comparisons were made using Two-Way repeated-measures ANOVA with Bonferroni's *post-hoc* test. Data represent mean ± S.E.M. from 3 independent experiments with the average value from each experiment being *n* = 1.

Biochemical analyses of astrocytes cultured on different substrates were undertaken in the absence and presence of drug treatments. Cell viability [3-(4,5-dimethylthiazol-2-yl)-2,5-diphenyltetrazolium bromide assay] and cell damage (lactate dehydrogenase assay) revealed no significant detrimental effects of treatments (Figure 4). Minor but significant increases in cell viability or decreases in cell damage were observed in some combinations of substrate and drug treatment, but no obvious patterns were apparent. In all cases where significant changes were observed, drug treatments (Fasudil or Y27632) either increased mitochondrial activity or decreased cell damage; detrimental changes were not observed following drug treatments.

## Discussion

Given astrocytes are a plastic glial population existing in various morphologies and displaying diverse biologies, major and wide-ranging effects would be expected upon manipulation of the ROCK/Rho system, a key determinant via actin of cellular survival, migration and proliferation (Riento and Ridley, 2003). Whilst our initial bioinformatic analyses of transcriptomic changes induced by Fasudil were directed at major biological processes regulating cytoskeletal reorganization, we also found significant changes in expression of a diverse group of genes associated with astrocyte function, and suggested that overall ROCK inhibitors would produce “healthy and physiologically beneficial responses in astrocyte biology” (Lau et al., 2012). We thus postulated the existence of a pro-survival, cytotrophic phenotype wherein the essential criteria were a preponderance of G-actin and elevated expression of EAAT2, BDNF and key anti-oxidant genes. Reappraisal of our bioinformatic data revealed diverse additional effects on the astrocyte transcriptome likely to be beneficial in brain injury. Gene expression profiles of motor and autophagic cellular cascades and inflammatory/angiogenic responses were all inhibited favoring adoption of what might be considered “healthy,” anti-migratory phenotype. These types of changes generally have not been a focus in astrocytes, although often documented in neurones, but there is the beginnings of an new literature describing roles for Rho GTPases or actions of ROCK inhibitors in the biological processes reported here (*vide supra*). This concept of an anti-migratory phenotype (Cárdenas et al., 2014) is an interesting one for *in vitro* ROCK inhibitors produced very rapid stellation of astrocytes (approximately 15 min with most changes complete by 3–6 hr), a morphology which in uninjured brain is considered to reflect non-migratory properties, and here may indicate Fasudil produces a normal cytotrophic astrocyte. This state may resemble cytotrophic components of minimal, self-resolving hypertrophy often stated to occur in minor trauma/injury wherein there is re-establishment of a healthy physiological phenotype (Balasingam and Yong, 1996; Sofroniew, 2009; Burda and Sofroniew, 2014). Earlier work has documented the ability of ROCK inhibitors to produce extension of GFAP-positive, presumed astrocytic processes *in vitro* and *in vivo* models of nerve crush (Sagawa et al., 2007; Ichikawa et al., 2008). Extensive process formation has also been noted with ROCK inhibitors in wound healing models using astrocytes (Holtje et al., 2005). Such morphological rearrangements as discussed here are underpinned by extensive remodeling of the actin cytoskeleton, particularly lamellipodia and filopodia (Le Clainche and Carlier, 2008; Mattila and Lappalainen, 2008).

Tissue engineering in combination with materials science has been also been employed to manipulate astrocyte biology. We have previously reported in an *in vivo* model of traumatic brain injury (Nisbet et al., 2009) that, in the presence of PCL scaffolds, astrocytes provided signals encouraging neuritic infiltration of injured tissue, with findings favoring the existence of early cytotoxic and late cytotrophic components of astrogliosis. Others have reported variable data with astrocytes in tissue engineering and the literature suggests that the type of engineering approach employed greatly influences the nature of the outcome, and specifically the temporal contributions of cytotrophic vs. cytotoxic astrogliosis (Iannotti et al., 2003; Wong et al., 2007; Nisbet et al., 2010). Nevertheless our success *in vivo* encouraged us to pursue mechanistic studies with tissue engineering of astrocytes on PCL bioscaffolds where we found in 3D extensive process formation, stellation and adoption of a cytotropic phenotype resembling that found in 2D astrocytes treated with ROCK inhibitors (*vide supra*). There have been remarkably few successes where bioengineering strategies *in vitro* have led to the establishment of viable astrocytes on bioscaffolds (Puschmann et al., 2013; Zuidema et al., 2014), but our use of secondary astrocytes allowed the long term maintenance of mature cells on PCL bioscaffolds (Lau et al., 2014). Given there was a body of evidence for beneficial effects of ROCK inhibitors in models of head and spinal trauma (Raad et al., 2012; Watzlawick et al., 2014) we extended our study to explore whether the inclusion of Fasudil and Y27632 would provide further benefits in our 3D astrocyte model. Here we found that both ROCK inhibitors produced additional GFAP-positive processes relative to PCL scaffolds alone, and there seemed a bonus of a further shift to an even greater preponderance of G-actin relative to F-actin. AHNAK, a marker of enlargeosome activity and migration (Racchetti et al., 2012), proved very suitable for immunostaining of fine astrocytic processes, being partially co-localized with GFAP, but revealing much more of the astrocyte arbor than GFAP, an effect which was particularly obvious on aligned bioscaffolds. Overall these data are consistent with ROCK inhibitors providing further beneficial effects over and above PCL scaffolds alone. Preliminary evidence from Western immunoblotting suggested ROCK inhibitors reduced GFAP expression relative to dbcAMP (Supplementary Figure 1), whilst patterns of AHNAK expression were generally consistent with immunocytochemistry. In ongoing work examining Glu transporter activity, we confirmed our previously reported elevation of uptake in 2D astrocytes by Fasudil and Y27632 (Lau et al., 2011), and found EAAT activity appeared to be elevated 2–4 fold in astrocytes maintained on bioscaffolds (data not shown). Whilst we need to undertake further experiments to document fully the phenotype of the astrocytes found here, it does seem that the phenotype may be shifted even further toward the direction of cytotrophic astrogliosis. Our findings here, demonstrating that Fasudil and Y27632 under certain conditions either increased cell viability or decreased cell damage compared with control conditions (Figure 4), provide further evidence for the potential benefits of ROCK inhibitors via direct effects on astrocytes, which may contribute to the beneficial outcomes of these treatments in brain injury. Taken together there would seem to be a case for combining Rho kinase inhibition with tissue engineering in models of traumatic brain and spinal cord injury.

ROCK inhibitors, and especially Fasudil, have been examined in various injury models where astrocytes may contribute to the pathology, perhaps via cytotoxic inflammation, and/or by compromising recovery from trauma/neurodegeneration. Glia, including astrocytes, are known to contribute to the neuropathology of MND through non-cell autonomous mechanisms (Vargas and Johnson, 2010; Pirooznia et al., 2014). MND is a rapidly advancing degenerative condition where suitable new therapeutic strategies are badly needed (Turner and Talbot, 2008), so the pro-survival response produced by Fasudil in the SOD1 mouse model of MND is an impressive advance (Takata et al., 2013; Tonges et al., 2014) since the rapid progression of disease in this model generally does not respond to interventions (Turner and Talbot, 2008). Attentuation of astroglial pathology was noted early after Fasudil treatment whereas beneficial effects were noted at most stages on microglial mediated inflammation (Tonges et al., 2014). Rho kinase inhibitors may also be useful in other neurodegenerative conditions where astrocytes contribute to inflammation, perhaps by non-cell autonomous mechanisms. For example in Alzheimer's disease, where astrocytic mechanisms are linked to disease risk factors and where their contribution to synaptic signaling is compromised by amyloid-β peptide (Talantova et al., 2013), Fasudil may have therapeutic potential as it suppressed the inflammation in rodent hippocampus induced by amyloid-β peptide (Song et al., 2013). In experimental autoimmune encephalomyelitis, an animal model of multiple sclerosis, Fasudil reduced inflammation and demyelination. Early or late administration of Fasudil exerted beneficial effects producing a shift in macrophage function from the cytotoxic M1 to the anti-inflammatory or reparative M2 phenotype in spinal cord and spleen (Liu et al., 2013). Subsequently the same laboratory suggested that at least part of this action of Fasudil was via a diminution of cytotoxic astrogliosis and less infiltration of inflammatory cells across the blood brain barrier (Guo et al., 2014). Interestingly, astrocyte phenotype is now recognized to determine the outcome of CNS repair and myelination, and components of astrocyte biology likely represent valid targets to enhance lesion repair in multiple sclerosis (Barnett and Linington, 2013).

Given the seminal role astrocytes play in synaptic transmission and maintenance of brain function generally, their diverse biology offers many options for potentially “druggable” targets—beneficial shifts to cytotrophic phenotypes would improve their overall health and ameliorate cytotoxic inflammation in brain pathologies, and thus conceivably allow maintenance of appropriate synaptic function (Vargas and Johnson, 2010). Another often not discussed aspect of astrocyte biology is their multiple “morphological” interfaces, not only via the communication of astrocytic tight junctions, but also with different cellular populations viz. neurones, oligodendrocytes, blood vessels, blood brain barrier and microglia (Volterra and Meldolesi, 2005), including via the quad-partite synapse (Schafer et al., 2013). Thus astrocytes are seminally placed from an organizational perspective to orchestrate the biology of these different CNS populations by integrating synaptic and non-synaptic signaling. The recent work with ROCK inhibitors showing cytotrophic changes in astrocyte function in disease models of MND (Tonges et al., 2014) and multiple sclerosis (Guo et al., 2014) are particularly encouraging. Indeed, we speculate that Fasudil-induced changes in astrocytic phentotype exert beneficial effects which should be taken in a similar context to the much popularized “healthy” shift in macrophage function (now extended to microglia) from the cytotoxic M1 to the reparative M2 phenotype—and which is produced by Fasudil in spinal cord in experimental autoimmune encephalomyelitis (Liu et al., 2013). Meta-analysis of data from experimental studies of spinal cord injury evaluating Rho A/ROCK blockade found significant overall improvement of locomotor function. Overall a possible role in inflammatory events was noted and the strategy was considered a plausible one for management of human spinal cord injury (Watzlawick et al., 2014). Certainly it is clear with advances in the design of increasingly effective ROCK inhibitors (Guan et al., 2013), and with new developments in tissue engineering and drug delivery via nanoparticles that ROCK inhibitors alone or in concert with these new technologies are likely to be widely applicable to management of inflammation in neurodegenerative conditions.

### Conflict of interest statement

The authors declare that the research was conducted in the absence of any commercial or financial relationships that could be construed as a potential conflict of interest.

## Abbreviations

CNS: central nervous system
dbcAMP: N^6^,2′-O-dibutyryladenosine 3′,5′-cyclic monophosphate
DRP2: dystrophin related protein 2
EAAT: excitatory amino acid transporter
ECM: extracellular matrix
FOXO: mammalian forkhead members of class O
Glu: L-glutamate
HIF-1: hypoxic-inducible factor 1
KIF: kinesin family member
MFN1: mitofusin 1
MND: motor neurone disease
NFKB1: nuclear factor of kappa light chain gene enhancer in B cells 1
PCL: poly-ε-caprolactone
RAB: member of RAS oncogene family
RHOT1/2: ras homolog gene family members T1/2
ROCK: Rho kinase
SOCS3: suppressor of cytokine signaling 3
SQSTM1: sequestosome 1
STX1A: syntaxin binding protein 1A
VEGF: vascular endothelial growth factor.
