# Supplementary material for: Transcriptomic analysis and 3D bioengineering of astrocytes indicate ROCK inhibition produces cytotrophic astrogliosis
Source: Front Neurosci. 2015 Feb 20;9:50. doi: 10.3389/fnins.2015.00050 (PMC4335181; doi:10.3389/fnins.2015.00050)
Supplement: Supplementary file 1 [file DataSheet1.PDF]

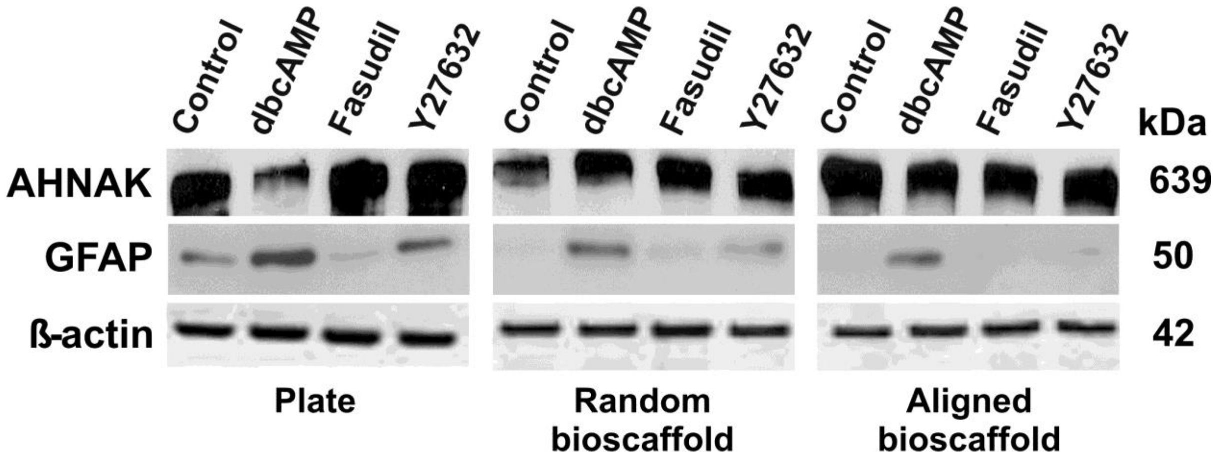

Supplementary Figure 1. Western blots of GFAP and AHNAK expression from astrocytes on culture plates (2D), random and aligned bioscaffolds. Astrocytes were treated with dbcAMP (1 mM), Fasudil (100 µM) or Y27632 (30 µM) for 72 h. Procedures have been described previously (Lau et al. Br J Pharmacol 163: 533-545 (2011) and employed ~10 µg protein with MW markers and ß-actin (MW 42 kDa) was used as a loading control.
